# Increased expression of Id1 and Id3 promotes tumorigenicity by enhancing angiogenesis and suppressing apoptosis in small cell lung cancer

**DOI:** 10.18632/genesandcancer.20

**Published:** 2014-05

**Authors:** Danqing Chen, Shiva S. Forootan, John R. Gosney, Farzad S. Forootan, Youqiang Ke

**Affiliations:** ^1^ Molecular Pathology Laboratory, Department of Molecular and Clinical Cancer Medicine, Liverpool University, 5/6th Floor, Duncan Building, Daulby Street, Liverpool, L69 3GA, UK.

**Keywords:** Id1, Id3, Small Cell Lung Cancer, Tumorigenicity, Angiogenesis, Apoptosis

## Abstract

Constant deregulation of Id1 and Id3 has been implicated in a wide range of carcinomas. However, underlying molecular evidence for the joint role of Id1 and Id3 in the tumorigenicity of small cell lung cancer (SCLC) is sparse. Investigating the biological significance of elevated expression in SCLC cells, we found that Id1 and Id3 co-suppression resulted in significant reduction of proliferation rate, invasiveness and anchorage-independent growth. Suppressing both Id1 and Id3 expression also greatly reduced the average size of tumors produced by transfectant cells when inoculated subcutaneously into nude mice. Further investigation revealed that suppressed expression of Id1 and Id3 was accompanied by decreased angiogenesis and increased apoptosis. Therefore, the SCLC tumorigenicity suppression effect of double knockdown of Id1 and Id3 may be regulated through pathways of apoptosis and angiogenesis.

## INTRODUCTION

The incidence of lung cancer has been rising since the 1930s. It is the second most common malignancy in both men and women (around 14% of all new cancers) and has been the leading cause of cancer deaths in the US among men since 1950s and among women since 1987 (1). SCLC is the most aggressive subtype of lung cancer and accounts for approximately 15-25% of all malignant tumors of the lung (2). Despite a generally good initial response to chemotherapy and radiotherapy, SCLC has a poor prognosis due to its rapid dissemination. The 5-year survival rate is 10-13% for limited-stage (LS-SCLC) and only 1-2% for extensive-stage (ES-SCLC) disease (3).

Crucial genetic alterations must clearly contribute to the tumorigenicity of SCLC cells, but despite the large number of investigations into the potentially important molecular events both in the laboratory and in pre-clinical trials, novel therapies for the disease have served only as adjuncts to routine chemotherapy, rather than as primary treatments, due to either “off-target” effects or lack of adequate biological activity (4-6). Our understanding of the key factors in the tumorigenicity of SCLC is limited. In our previous study, gene expression profiles were compared between the normal bronchial cell line Beas-2B and a malignant SCLC cell line Lu-165 using the microquantity differential display technique (MDD) developed in our laboratory (7). A cDNA fragment that had 100% homology to the gene coding for Id1 was identified to be up-regulated (8). The Id proteins possess a helix-loop-helix domain but lack a DNA-binding domain, thereby functioning as dominant negative regulators of basic HLH (bHLH) transcriptional regulators in numerous cellular processes including differentiation, proliferation, metastasis, apoptosis, angiogenesis, and neoplastic transformation (9, 10). Overexpression of Id1 and Id3 has been correlated with an unfavorable prognosis in a variety of cancer types (11-13) and a recent study suggested Id1 and Id3 co-expression was related to poor clinical outcome in stage III-N2 non-small cell lung cancer (NSCLC) (14). In addition, Id1 and Id3 are co-expressed temporally during murine neurogenesis and angiogenesis (15, 16), a single copy of Id1 or Id3 being sufficient to rescue embryonic angiogenesis in the brain, but not neovascularization of tumors (17).

In contrast to studies in other malignancies, including NSCLC, the possible roles of Id proteins in SCLC have not been explored and the biological significance of the elevated expression of Id1 and Id3 remains elusive, although they were overexpressed in both cell lines and biopsy specimens compared with their benign counterparts (8). In this study, we first established SCLC cell line N417-derived sublines expressing reduced levels of Id1 and Id3 by transfection of a single vector constructed to co-express two shRNAs simultaneously. Then we investigated the effect of either singly or jointly suppressed Id1 or Id3 on tumorigenicity of SCLC cells *in vitro* and *in vivo*. The molecular mechanisms involved in their functional roles and the potential of targeting Id1 and Id3 as methods of inhibiting malignant progression of SCLC cells were also assessed.

## RESULTS

### Identification of the most effective suppressor against Id1

**Figure 1:**
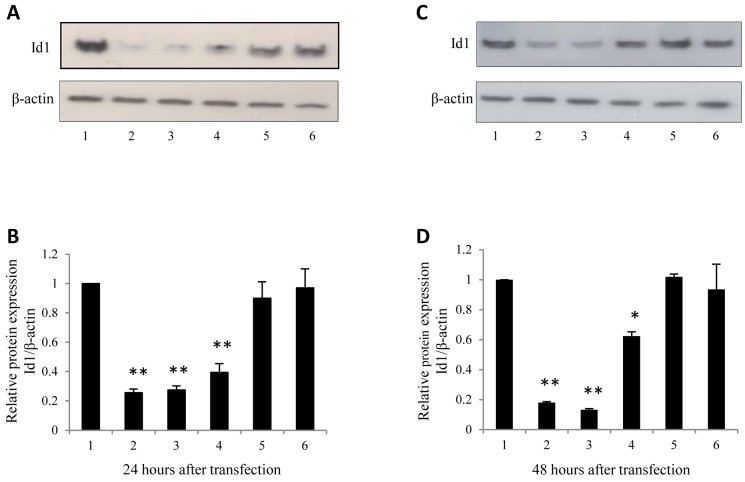
Western blot analysis of the Knockdown effect of 3 candidate siRNAs on Id1 expression at protein level SCLC cell line N417 was used as RNA recipient cells. The levels (shown in A, B, C and D) of Id1 expressed were measured in control cells and in cells transiently transfected with different siRNAs: 1, untreated N417 cells; 2, N417+ siRNA1; 3, N417+ siRNA2; 4, N417+ siRNA3; 5, N417+ scramble RNA; 6, N417+ transfection reagent. An antibody against β-actin was also incubated with the blot to normalize the possible loading errors. (A). Western blot analysis of Id1 expressed in the control cells and in the cells transfected transiently with different RNAs for 24 hours. (B). Quantitative analysis of Id1 levels detected with Western blot. The level of Id1 protein expressed in the parental N417 cells was set at 1; levels of Id1 expressed in cells after 24 hours of different transient transfections were calculated by relating to that in the parental cells. (C). Western blot analysis of Id1 expressed in the control cells and in the cells transfected transiently with different RNAs for 48 hours. (D). Quantitative analysis of Id1 levels detected with Western blot. The level of Id1 protein expressed in the parental N417 cells was set at 1; levels of Id1 expressed in cells after 48 hours of different transient transfections were calculated by relating to that in the parental cells. Id1 protein relative expression levels after either 24 or 48 hours were quantified by scanning the intensity of band areas on the blot through densitometry and normalized to β-actin. The results were obtained through 3 separate measurements and the statistical difference was determined by 2-tailed Student's *t*-test (**P<0.0001).

The 3 selected siRNAs were subjected to transient transfection to test their capability of silencing Id1 and Id3 in SCLC cell line N417 and to identify the optimal silencer. The effect of Id1 suppression produced by the 3 siRNAs was shown in Fig. 1. At 24h after the transient transfection, all 3 siRNAs produced a reduction in Id1 level (Fig. 1A), but both scramble RNA and transfection reagent controls did not significantly change the Id1 expression level. When the Id1 level in the parental cells was set at 1, the levels produced by transfections with siRNA1, siRNA2 and siRNA3 were significantly reduced to 0.26, 0.27 and 0.39, respectively (Fig. 1B, *P*<0.0001). Although further suppression was obtained at 48h after the transient transfection with siRNA1 and siRNA2, the suppression caused by siRNA3 was reversed partially (Fig. 1C). While Id1 levels at 48h after the transient transfection with siRNA1 and siRNA2 were reduced to 0.18 and 0.13 respectively (Fig. 1D, *P*<0.0001), the level after siRNA3 transient transfection was reduced to 0.62 (Fig. 1D, *P*<0.0001). Thus combined the suppression effects in both experimental time points (24 and 48h), siRNA2 which produced a 3.9- and 7.7-fold reduction respectively, was chosen as the optimal siRNA sequence and used for further study. The optimal Id3 siRNA was previously selected in a similar manner (18).

### Establishment of stable transfectants expressing attenuated levels of Id1 and Id3

**Figure 2:**
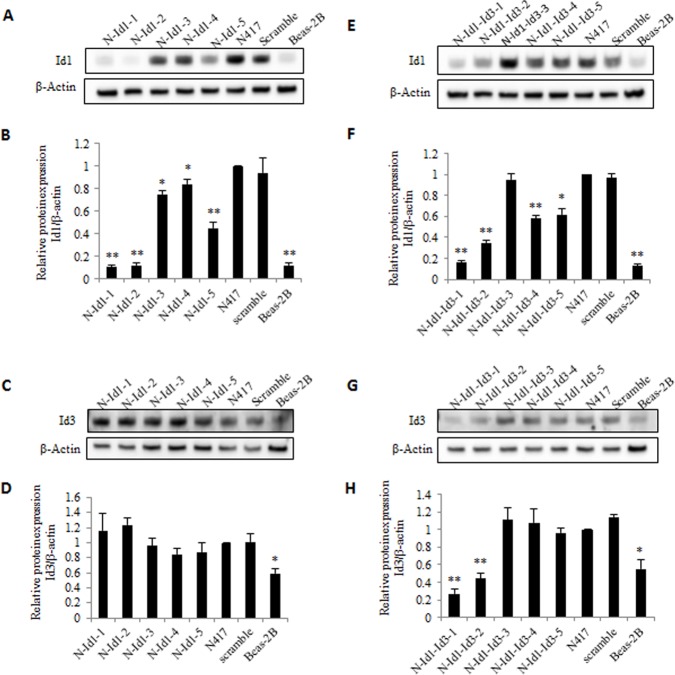
Knockdown effect of stable transfection of shRNAs targeting either Id1 and Id3 or Id1 alone on levels of Id1 and Id3 or on that of Id1 expressed in N417 cells Five individual sublines were generated from five different individual shRNA transfectant clones. The parental benign Beas-2B bronchial epithelial cells and the transfectants generated by a plasmid harboring scramble RNA only were used as controls. (A). Western blot analysis of Id1 expression in five sublines generated from clones of Id1-shRNA transfectants. (B). Relative Id1 levels in the five Id1-shRNA -transfected sublines. (C). Western blot analysis of Id3 expression in the five Id1-shRNA-transfected sublines. (D). Quantitative analysis of relative Id3 levels in the five Id1-shRNA-transfected sublines. (E). Western blot analyses of levels of Id1 expression in the five sublines established from separate clones of Id1- and Id3- shRNA transfectants. (F). Relative levels of Id1 expressed in the five Id1- and Id3- shRNA- transfectant sublines. (G). Western blot analysis of Id3 expression in the five Id1- and Id3-shRNA- transfectant sublines. (H). Relative levels of Id3 expressed in the five Id1- and Id3- shRNA- transfectant sublines. For all quantitative measurements, the level of Id1 or Id3 expressed in control N417 cells was set at 1; levels in other transfectant lines were calculated by relating to that expressed in the control cells. Results were presented as the means and SD from three independent experiments. Statistical difference was determined by 2-tailed Student's *t*-test (**P<0.0001, *P<0.05).

The expression of Id1 and Id3 was knocked down either singly or jointly in the 5 sublines established from each transfectant group. The expression levels of Id1 and Id3 in the 2 transfectant groups (each had 5 sublines) were measured by Western blot and were shown in Fig. 2. The Id1 expression levels were reduced unevenly amongst the five Id1 shRNA transfectant cell lines in comparison with the parental N417 cells (Fig. 2A). When the Id1 level in the N417 cells was set at 1, the Id1 levels in N-Id1-1, N-Id1-2 and N-Id1-5 were significantly reduced to 0.1, 0.12 and 0.45, respectively (Fig. 2B, *P*<0.0001). The relative expression of Id1 in N-Id1-1 and N-Id1-2 was reduced to levels similar to that expressed in the benign Beas-2B cells (0.12). Comparing to that in the parental cells, Id1 protein levels in N-Id1-3 and N-Id1-4 were decreased to 0.75 and 0.84 respectively. The Id3 expression in all Id1 shRNA transfectant cell lines was detected with Western blotting (Fig. 2C) and its relative levels in those cells were ranged from 0.85 to 1.24, which was not significantly different from the parental N417 cells (Fig. 2D) (*P*>0.05). Likewise, the Id1 levels in five Id1 and Id3 double knockdown cell lines were all inhibited, but with a degree of difference (Fig. 2E). While the levels of Id1 protein in N-Id1-Id3-1, N-Id1-Id3-2 and N-Id1-Id3-4 were high-significantly suppressed to 0.16, 0.35 and 0.58, respectively (Fig. 2F, *P*<0.0001) in comparison to the control, the Id1 relative level in N-Id1-Id3-5 and in N-Id1-Id3-3 was only slightly reduced to 0.62 and 0.95, respectively. The level of Id1 in N-Id1-1 cells was similar to that in the benign Beas-2B cells (0.13). Although levels of Id3 were greatly reduced in 2 double knockdown transfected cell lines, there was nearly no RNAi silencing effect on other 3 cell lines (Fig. 2G). The relative levels of Id3 protein in N-Id1-Id3-1 and N-Id1-Id3-2 were further reduced to 0.27 and 0.44 (*P*<0.0001), even lower than that in Beas-2B (0.55) (Fig. 2H). Thus one highly suppressed cell line (N-Id1-1, N-Id1-Id3-1) and one moderately suppressed cell line (N-Id1-5, N-Id1-Id3-2) from each group of transfectant cell lines were selected for further analysis.

### Effect of knockdown of Id1 alone or Id1 and Id3 jointly on malignant characteristics of cells

**Figure 3:**
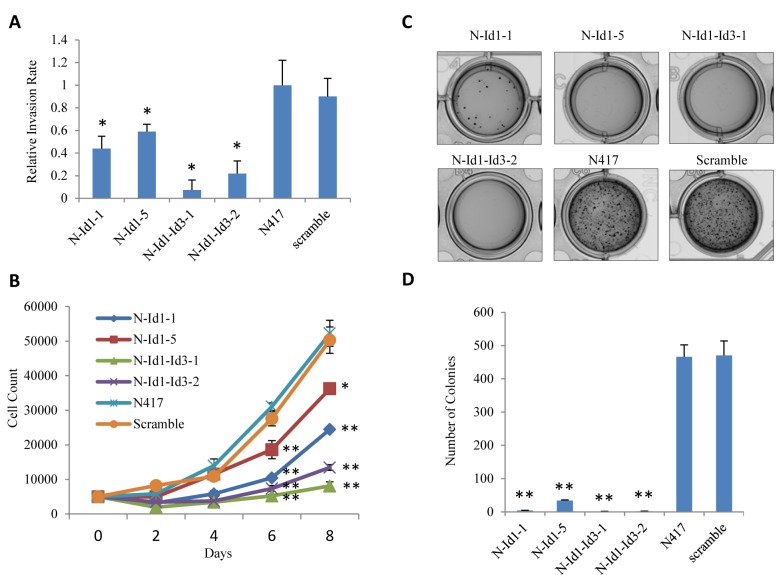
Suppressive Effect of transfection of Id1- and Id3-shRNAs jointly or Id1-shRNA alone on invasiveness and proliferation of N417 cells Sublines, in which the target gene products were highly (N-Id1-1, N-Id1-Id3-1) and moderately (N-Id1-5, N-Id1-Id3-2) suppressed, were identified by either Id1 and Id3 levels or the Id1 level in those five transfectant sublines from clones derived from either joint Id1- and Id3-shRNA transfectants, or from Id1-shRNA alone transfectants. (A). The relative invasiveness of different transfectants was assessed by a transwell invasion assay. Invasiveness of the control N417 cells was set at 1. (B). Proliferation rates of different transfectant cells were tested by a proliferation assay performed in 96-well plates for a period of eight days. (C). Soft agar assay was performed to test the nodule-forming ability of different transfectant cells. A representative soft agar well from each test group displayed in the photograph was taken at the end of 6 week inoculation period. (D). Number of colonies produced by different transfectant cells in soft agar. Results (mean and SD) were obtained from the triplicated wells. The difference between the colony numbers produced by each transfectant cells and those by the control cells was compared by 2-tailed Student's *t*-test (**P<0.0001, *P<0.05).

The effect of transfection of Id1- and Id3- shRNA jointly or Id1- shRNA alone on invasiveness, proliferation and anchorage-independent growth of the cells was shown in Fig. 3. The invasiveness of different transfectant cells was shown in Fig. 3A. When the percentage rate of the invaded parental N417 cells was set as 1, the relative invasion rate of N-Id1-1 and N-Id1-5 was significantly reduced to 0.44 and 0.59 respectively (*P*<0.04, Fig. 3A). The relative invasiveness in double knockdown transfectant cells N-Id1-Id3-1 and N-Id1-Id3-2 was further significantly reduced to 0.07 and 0.22 respectively (*P*<0.007, Fig. 3A). The relative invasiveness of scramble control (0.9) was not significantly changed from that of the parental cells (*P*=0.78). The result of cell proliferation assay was shown in Fig. 3B. While the changes on proliferation rate did not exhibit significant difference on day 2 and day 4, numbers of cells from N-Id1-1, N-Id1-5, N-Id1-Id3-1 and N-Id1-Id3-2 on day 6 decreased to 10467±782, 18607±2585, 5233±411 and 7382±850, a 3-, 1.7-, 5.9- and 4.2-fold significant reduction (*P*<0.002) respectively, in comparison with that of the parental control. Further changes were observed on day 8. Numbers of cells reduced to 24483±797, 36269±1206, 8100±1175, and 13463±834 in N-Id1-1, N-Id1-5, N-Id1-Id3-1, and N-Id1-Id3-2, a reduction of 3.1-, 1.4-, 6.4- and 3.9-fold respectively (*P*<0.003). No significant difference between parental cells and scramble controls was detected either on any time points prior to day 6, or on day 6 and day 8 (*P*=0.11, *P*=0.06, *P*=0.07, *P*=0.56). The results of the anchorage-independent growth capability of the cells (as an indication of tumorigenicity) or soft agar assay were shown in Fig. 3D. After 4-6 weeks, the number of colonies produced by N-Id1-1 and N-Id1-5 was dramatically decreased by 116.5- and 13.7- fold to 4±0.57 and 34±1.76 respectively, as compared with the parental cells (466±36.12) (*P*<0.0001). Impressively, very small, and similar numbers of colonies were produced by the N-Id1-Id3-1 and N-Id1-Id3-2 cells (1±0.57 and 2±0.57, respectively), high significantly reduction to 366- and 233- fold, respectively (*P*<0.0001). No significant difference was observed between the parental and scramble controls (*P*=0.743).

### Inhibition of Id1 and Id3 greatly suppressed the tumor growth in nude mice

**Figure 4:**
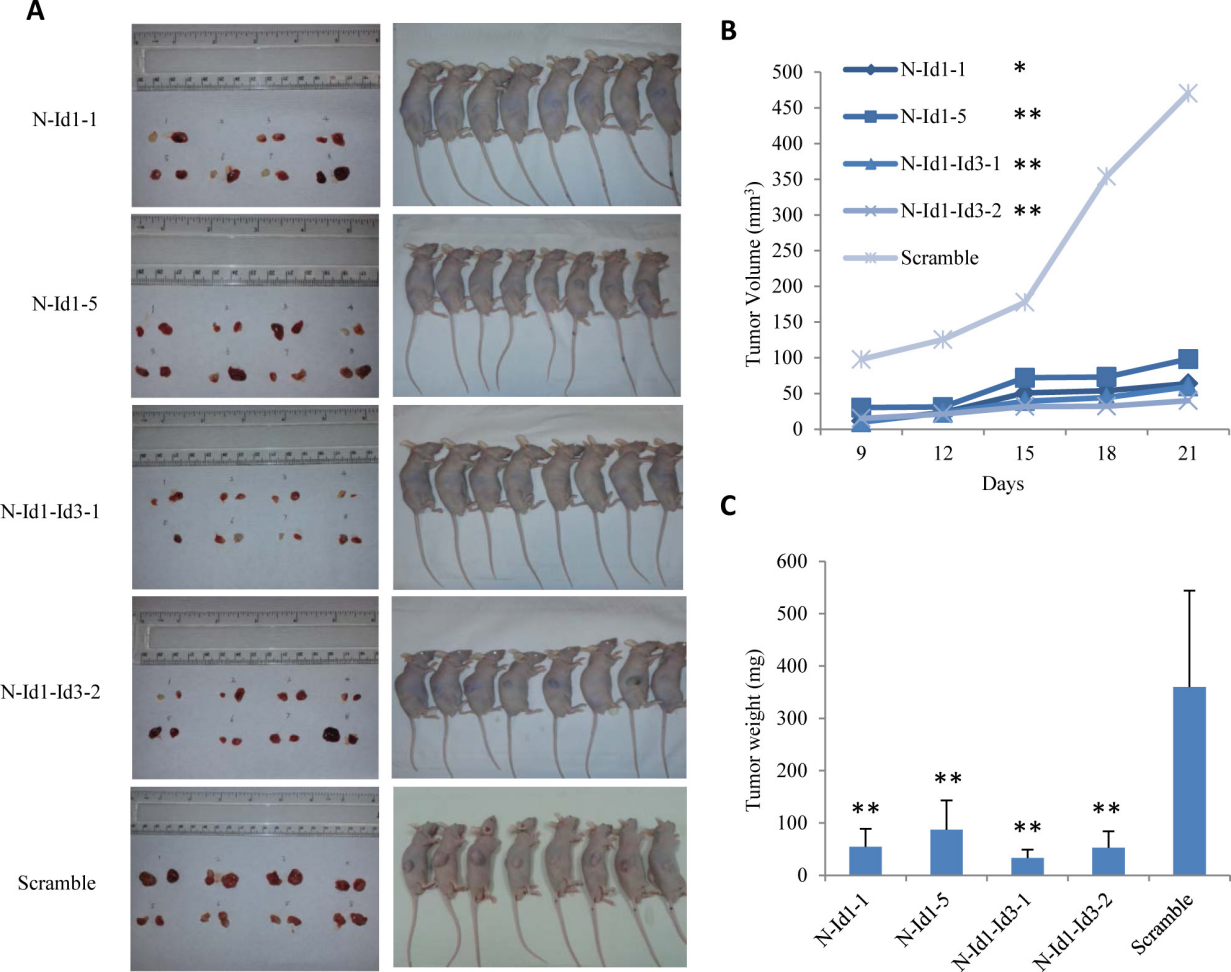
Testing tumorigenicity of different transfectant cells in nude mouse Transfectant cells N-Id1-1, N-Id1-5, N-Id1-Id3-1, N-Id1-Id3-2 and the control (scramble RNA transfected) cells were inoculated into both flanks of male BALB/c nude mice to produce tumors. (A). Tumor produced in control and each of the 4 testing groups. Left panel: tumors resected at autopsy (21 days after the initial inoculation); Right panel: different groups of mice that bear tumors with different sizes (21 days after the initial inoculation). (B). Average volume of tumors produced by each group of animals at different time points in a 21 day experimental period. (C). Average weight of tumors produced by each group of animals at the autopsy (21 days after the inoculation). The mean weight of tumors produced by each group of mice was compared with that by the control group by 2- tailed Student's *t*-test (n=8, **P<0.0001).

**Figure 5:**
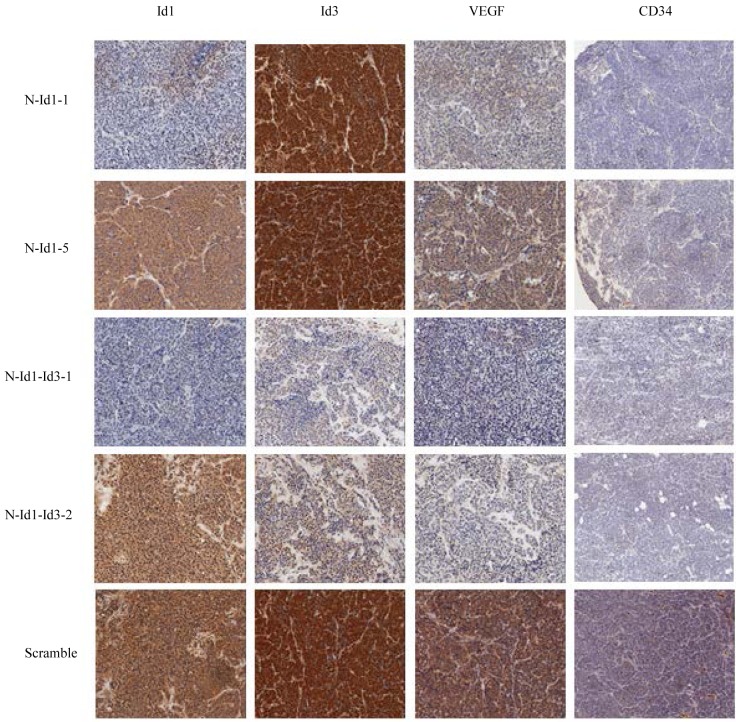
Immunohistochemistry analysis of the expression status of Id1, Id3, VEGF, and the microvessel densities (by CD34 antibody) of tumors produced in mice Sections of tumor samples from each group were immunohistochemically stained with antibodies against Id1 (left column), Id3 (column 2), VEGF (column 3), and CD34 (right column), respectively. The staining intensities with different antibodies were scored by analyzing ten fields per slide. Original magnification: 10×.

To test the effect of inhibiting Id1 and Id3 expression in SCLC, Id1- and Id3- shRNA transfectant cells were inoculated in nude mice and the tumors produced by different transfectants were measured at different time points and resected at autopsy. Tumors resected from different groups of mice and the average sizes of tumors produced by the control and testing groups were shown in Fig. 4. Nude mice in all groups gained weight over the course of the study. All tumors were visualized 8 days after the inoculation thus no significant difference was seen on lengths of latent periods. The scramble RNA transfected cell group elicited more rapid tumor growth than the N-Id1 and N-Id1-Id3 groups (Fig. 4A). In N-Id1-1 group, 1 mouse failed to produce tumors in either of the 2 flanks. In N-Id1-Id3-1 group, 1 mouse failed to produce tumor in the left flank. As a whole, no significant differences in tumor incidences were tested between the control and each of the testing groups (*P*=0.48, *P*=1). As shown in Fig. 4B, the average sizes of tumors produced by the control group on day 9, 12, 15, 18 and 21 respectively were all significantly larger than those produced by N-Id1-1, N-Id1-5, N-Id1-Id3-1 and N-Id1-Id3-2 groups at the same time-points (*P*<0.0001). At autopsy, the average volumes of tumors produced by N-Id1-1, N-Id1-5, N-Id1-Id3-1 and N-Id1-Id3-2 were 63±32, 98±49, 60±14 and 40±28 mm^3^ respectively; 7.8-, 5-, 12.3- and 8.2-fold significant reduction (*P*<0.0001) in compared with that produced by the scramble control (490±241 mm^3^). At the end of the experiment, the average weight of tumors produced in N-Id1-1, N-Id1-5, N-Id1-Id3-1 and N-Id1-Id3-2 groups were 55±34, 87±56, 33±16 and 53±32 mg, significantly reduction (*P*<0.002) of 6.6-, 4.1-, 10.9-, and 6.8-fold; respectively, in comparison with that produced by the control group (360±164 mg) (Fig. 4C). Immunohistochemical analyses of the expression status of Id1, Id3 and VEGF proteins and the microvessel density (stained by CD34 antibody) were performed in tumors produced by different transfectant cells in nude mice and a representative staining sample taken from the control and each of the testing groups was shown in Fig. 5. Comparing with the control, staining intensities with Id1 antibody in tumors produced by both N-Id-1 and N-Id-5 cells were reduced, but not those of the Id3 antibody staining. Whereas in tumors produced by both N-Id1-Id3-1 and N-Id1-Id3-2 cells, staining intensities with both Id1 and Id3 antibodies were remarkably reduced. Although the staining intensities with VEGF antibody in all tumors were decreased, the reductions in tumors produced by N-Id1-Id3-1 and N-Id1-Id3-2 groups were more than those observed in tumors produced by N-Id1-1 and N-Id1-5 groups. When stained with CD34 antibody, tumors produced by scrambled control group exhibited the highest number of microvessels. In further quantitative analysis, the relative microvessel density of tumors produced by the scrambled control group was set as 1; those of tumors produced by N-Id1-1, N-Id1-5, N-Id1-Id3-1 and N-Id1-Id3-2 were 0.24, 0.38, 0.04 and 0.17, respectively. Thus the microvessel densities in tumors produced by these testing groups were significantly reduced by 4.1-, 2.6-, 29.2- and 5.8-fold (p<0.0001) respectively.

### Effect of Id1 &Id3 suppression on sensitivity of apoptosis induction and angiogenesis

**Figure 6:**
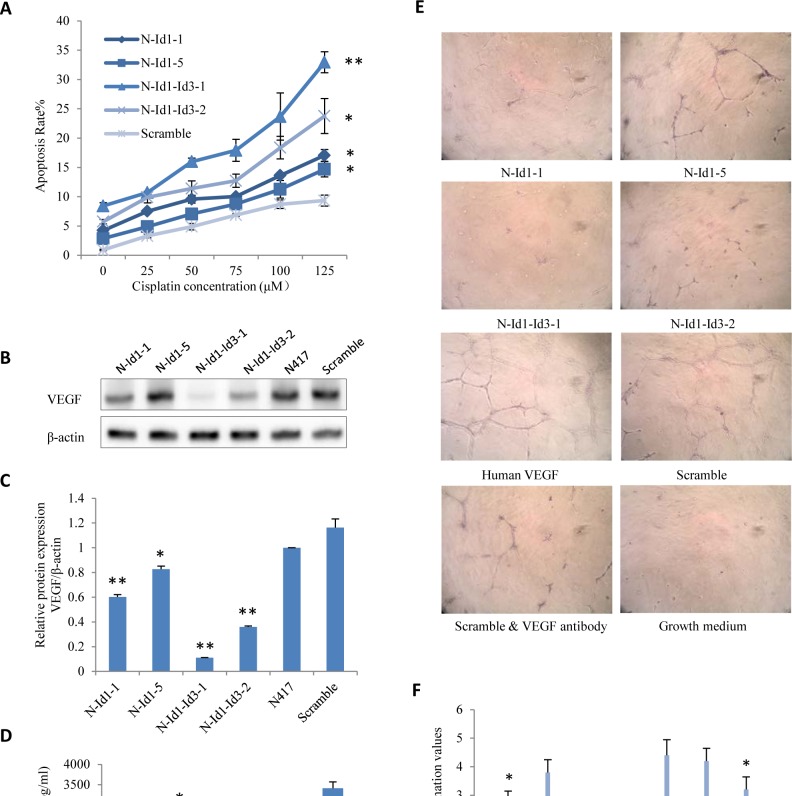
Effect of suppressing Id1 and Id3 or Id1 alone on cell apoptosis and angiogenesis (A). Induction of cell apoptosis with different doses of cisplatin. (B). Western blot analysis of VEGF expression in different shRNA transfectants, scramble RNA transfectants and the parental N417 cells. (C). Relative levels of VEGF expression in different cells. (D). ELISA analysis of concentrations of VEGF protein in the conditioned medium of different cells. (E). Measurement of biological activity of VEGF produced by different transfectant cells. Network formation observed by inverted microscope was in 40× magnification. (F). Scoring network formation produced by different conditioned media. Five randomly selected microscopic fields of each cell line under same condition were evaluated. **P<0.0001, *P<0.05.

The effect of suppressing Id1 and Id3 jointly or Id1 alone on sensitivity of apoptosis induction of SCLC and angiogenesis was shown in Fig. 6. The percentages of apoptotic cells in different cultured cell lines under the treatments with different doses of cisplatin (a first line drug used for chemotherapy) were shown in Fig. 6A. The percentage of cells undergoing apoptosis in the scramble control cells was 0.92%, whereas that in N-Id1-1, N-Id1-5, N-Id1-Id3-1, N-Id1-Id3-2 was 4.25%, 2.86%, 8.43%, 5.74% respectively. When treated with different dosages of cisplatin, the number of cells undergoing apoptosis was increased in a dosage-dependent manner in all treated cell lines. A low dose (25 μM) cisplatin treatment caused 3.3 to 10.7% of cells undergoing apoptosis in the control and other transfectant cells. Apoptotic cells increased as the increasing doses of cisplatin (Fig. 6A). When the highest dose of cisplatin (125 μM) was used, the percentages of the apoptotic cells remarkably induced to 17.06%, 14.69%, 32.94%, and 23.76% in N-Id1-1, N-Id1-5, N-Id1-Id3-1, and N-Id1-Id3-2 respectively, in comparison with that (9.34%) of the scramble control. Thus the number of apoptotic cells increased 1.6- to 3.6-fold in N-Id1 and N-Id1-Id3 compared to the control (*P*< 0.005). To assess the effect of changed expression levels of Id1 and Id3 on VEGF expression, Western blot and ELISA were conducted to detect VEGF expressed in the transfectant cell lines and that secreted into the culture medium. Results of Western blotting were shown in Fig. 6B, and more accurate quantitative analysis was shown in Fig. 6C. The VEGF protein levels in N-Id1-1, N-Id1-5 were mildly reduced to 0.6 and 0.83 respectively, while that in N-Id1-Id3-1 and N-Id1-Id3-2 were markedly reduced to 0.11, 0.36 respectively when compared with the control which was set at 1 (*P*<0.0004). The amount of VEGF in conditioned medium of cell culture collected from N-Id1-1, N-Id1-5, N-Id1-Id3-1 and N-Id1-Id3-2 were 1190±143, 2655±218, 297±28, and 554±30 pg/ml versus that in control (3415±155 pg/ml) (*P*<0.008) (Fig. 6D). To test whether the VEGF produced by different cells is biologically active, a HUVECs assay was performed to test the conditioned media and the results of the tube-like formations were shown in Fig. 6E. The network formation scores were shown in Fig. 6F. Conditioned medium of scramble control strikingly promoted endothelial tube formation, as well as human recombinant VEGF, with average score of 4.2 and 4.4 respectively. In contrast, N-Id1-1, N-Id1-5, N-Id1-Id3-1 and N-Id1-Id3-2 with average score of 2.6, 3.8, 1.4 and 2 respectively, significantly blocked the HUVECs forming branching networks (*P*<0.002).

## DISCUSSION

Id proteins are a group of transcription factors belonging to the basic helix-loop-helix (bHLH) family (19) and are involved in tumorigenesis of solid tumors (20, 21) and in T cell leukaemia (22). The biological function of bHLH proteins is to activate gene transcription by binding to each other forming homodimers or heterodimers which then enable them to bind the E-boxes (CANNTG) in the promoter region of corresponding genes (23). Id proteins act as negative dominant regulators of other bHLH factors by binding to these factors to inhibit them from making dimers and binding to E-box of DNA and, hence, to inhibit transcription (23) of important genes regulating the cell cycle (24, 25).

The effects of altering the binding potential of target genes by Id proteins are markedly different with different cell types (26). For example, Id2 may promote or inhibit tumorigenesis depending on the presence of E-proteins in host cells (27). Loss of Id2 can rescue the neurogenic and erythropoietic phenotype of *Rb*-null embryos but cannot ameliorate the severe muscle development defect (28). Loss of Id2 leads to a change of cell fate in mammary tissue that is not only associated with early differentiation, but inhibits proliferation of intestinal epithelia (29, 30). While loss of Id1 and Id3 leads to premature differentiation during embryogenesis (31), Id1/Id3 knockdown-inhibited differentiation and modulated tumorigenesis were reported in pancreatic and gastric epithelial cells (32, 33). Although Id1, Id2 and Id3 were highly over-expressed in SCLC in our previous work, it is not known what exact roles they may play in SCLC cells and whether they can be used as a joint or separate treatment targets for tumor suppression. In recent work, we demonstrated that suppression of Id3 expression in SCLC cells produced significant reductions in proliferation rates and in numbers of colonies formed in soft agar assay. When tested in nude mice, the final average volume of tumors produced by the Id3-suppressed cells was significantly reduced by 2- to 3- fold in comparison with the control. Upon induction of apoptosis by cytotoxin camptothecin, the percentages of apoptotic cells in Id3-suppresed cells were more than 2.4- fold higher than that in control. The reduction in tumor sizes achieved in this study was obtained by reducing Id3 only. The level of Id1 was not affected (18).

In this work we assessed whether Id1 and Id3 are synergistically promoting tumorigenicity of SCLC. As shown in our previous study (8), levels of Id1 and Id3 expression in the highly malignant SCLC cell line N417 were considerably higher than those detected in the benign Beas-2B cells. To study the effect of silencing Id1 and Id3 in N417, we identified the second siRNA as the optimal suppressor of Id1 through transient transfection. The optimal suppressor for Id3 was the same siRNA molecule as that used in our previous work (18). Then we introduced a single duet shRNA expression vector that facilitated the double knockdown of Id1 and Id3 in the N417 cells. Cloning two shRNAs into a single vector can minimize the risks of transfection times and increase the efficiency by avoiding additional shRNA vector construction, which is especially important for the cells with low transfection efficiency. In Id1-suppressed cells, the expression levels of N-Id1-1 and N-Id1-5 were reduced to 10% and 45% respectively (Fig. 2B) compared to parental cells, whereas the levels of Id3 in those cells were similar to the parental cells. This result suggested that suppressing Id1 expression did not change the expression level of Id3. This result was in line with our previous work which showed that shRNA transfection induced reduction of Id3 did not change the Id1 level. In Id1 and Id3 double knockdown cells, the expression levels of Id1 in N-Id1-Id3-1 and N-Id1-Id3-2 were reduced to 16% and 35% respectively, the levels of Id3 of those cells were 27% and 44% of that in parental cells (Fig. 2F, Fig. 2H). The Id3 suppression levels were similar as that in previous work (35% and 55%). Thus no cross effect was observed when Id1 and Id3 were suppressed by 2 separate shRNAs in the same cells simultaneously.

*In vitro* bioassay results showed that Id1-suppressed cells produced significant reductions in proliferation rate by 1.4- and 2.1-fold in comparison with the control (Fig. 3B). Our previous study showed that Id3-suppressed cells produced reductions in proliferation rates by 2.5- and 2.8-fold (18). Thus the efficiency of suppressing Id3 on cell proliferation was 1.3 and 1.8 times of that produced by suppressing Id1. The Id1 and Id3 double knockdown transfectants produced 3.9- and 6.4-fold reduction in cell proliferation rate when compared with the control (Fig. 3B). This result showed that the effect produced by joint suppression of Id1 and Id3 on cell proliferation was similar to, or more than the sum of the two effects produced by suppressing Id1 and Id3 separately. Soft agar assay results showed the number of colonies formed by Id1-suppressed cells was remarkably decreased by more than 13.7-fold compared with the parental cells (Fig. 3D). In Id3 suppressed cells, as shown in our previous work, an up to 3.7-fold reduction was produced in the number of colonies when compared to parental cells (18). Thus, suppression of Id1 produced nearly four times more suppression on anchorage-dependent growth of the cells than did suppression of Id3. The number of colonies produced by the two Id1 and Id3 double-knockdown transfectant cell lines (1±0.57 and 2±0.57, respectively) was dramatically reduced by 466- and 233- fold respectively when compared with the control (Fig. 3D). The reduction produced by joint Id1 and Id3 suppression was much larger than the sum of those produced by suppressing Id1 and Id3 separately. Thus, the reduced levels of Id1 and Id3 by combined suppression synergistically impaired the colony-forming ability of the SCLC cells in soft agar. The suppression effect was also observed in the invasion assay which showed that Id1-suppressed cells and Id1 and Id3 double knockdown cells produced more than 1.7- and 4.6- fold reduction respectively in relative invasiveness. Therefore, in three bioassays performed *in vitro*, targeting Id1 and Id3 simultaneously produced an at least equal, or more prominent effect than the sum of that produced by targeting Id1 or Id3 separately. These results obtained from SCLC cells are supported by earlier studies which revealed correlation of Id expression and cell proliferation, invasiveness and aggressiveness in other types of cancer (34-36).

When tested in nude mouse, the average volume of tumors produced in the Id1-suppressed group was significantly reduced by 5- and 7.5-fold, and that in Id1 and Id3 double knockdown was reduced by 8.2- and 11.8-fold (Fig. 4B). A similar reduction was observed by directly weighing the resected tumors at autopsy. The weight of tumor in the Id1-suppressed group was dramatically decreased by 4.1- and 6.6-fold, and that in the Id1 and Id3 double knockdown group by 6.8- and 10.9-fold (Fig. 4C). Our previous study showed that knockdown Id3 produced a reduction of 2.8- and 2.4-fold in average tumor weight and volume respectively (18). Taking all these results together, the greatest suppression of tumorigenicty was achieved by the double knockdown of Id1 and Id3 in a joint manner. Although this effect was significantly higher than each of the individual effects produced by knockdown of Id1 and Id3 separately, it was similar to the sum of those produced by separate knockdown of Id1 and Id3; no synergetic effect was observed.

Previous studies on breast cancer demonstrated that Id1 and Id3 promote malignant progression by inhibiting apoptosis of cancer cells (37). The results in our study showed that the number of apoptotic cells in Id1-suppressed transfectants increased by 1.6- to 1.8-fold. Our previous work showed that the percentage of cells undergoing apoptosis in Id3-suppressed transfectants was 2- to 3-fold higher than that of the control (18). In Id1 and Id3 double knockdown cells, the percentage of cells undergoing apoptosis increased by 2.5- to 3.5-fold (Fig. 6A). Thus, the greatest increment in the percentage of apoptotic cells was achieved through double knockdown of Id1 and Id3 in N417 cells. It was previously observed that Id proteins could induce apoptosis in some cell types (38-40), but also function as anti-apoptotic molecules in others (41, 42). Our results showed that inhibition of Id1 and Id3 promoted apoptosis of N417 cells in response to cisplatin treatment. While the percentage of apoptotic cells induced by cisplatin was increased in a dose-dependent manner, this increment was facilitated by the suppression of Id1/Id3 (Fig. 6A). This result suggested that Id1 and Id3 may be potential anti-apoptosis molecules whose increased expression may promote tumorigenicity of SCLC through, at least partially, suppressing apoptosis. The discovery in this study that suppression of Id1 and Id3 can increase the sensitivity of SCLC to cisplatin-initiated apoptosis-induction may provide new targets for designing more effective therapies for SCLC.

Some previous studies have demonstrated that both Id1 and Id3 promoted malignant progression of certain types of cancers through facilitating angiogenesis (43-47), a crucial feature of tumor development and progression (17, 31, 48, 49). In this study, it was observed that the level of VEGF was reduced with reduction of levels of Id1 and Id3 in SCLC *in vitro* and *in vivo* (Fig.6C, Fig. 6D, Fig. 5) and that reduced VEGF secreted by transfectant cells was associated with reduction of angiogenesis (Fig. 6F). Thus, the tumourigenicity-promoting activities of Id1 and Id3 in SCLC may be achieved, at least in part, by promoting angiogenesis. However, it was also noticed that the angiogenic activity of the N417 cells was not completely inhibited by the anti-VEGF antibody, indicating that, apart from VEGF, there may be other factors involved in promoting angiogenesis in the SCLC cells. The actual functional roles of Id1 and Id3 may be different and depend on the availability of E-Box proteins in a particular cell type (26). Analyzing the genomic sequence of VEGF gene with the Sequence Manipulation Suite, we found that there are eight “CAGCTG” and six “CACCTG” E-boxes in the promoter region. Thus the VEGF gene contains E-boxes. Id proteins preferentially dimerize with Group A members of E proteins and bind to these two E-box regions. The associative molecular events need further exploration. Earlier studies have shown that not only can Id1 and Id3 affect VEGF, but also that VEGF can influence Id1 and Id3. Thus, there may be a feedback loop between Id proteins and VEGF. A previous study showed that VEGF can stimulate Id1 and Id3 expression in HUVEC cells, and double knockdown Id1 and Id3 almost completely inhibited the proliferation and angiogenic processes which were induced by VEGF (50). It was speculated that VEGF is associated with activation of the MAPK cascade, which can induce the expression of Id proteins. The presence of VEGF in the circulation resulted in marked up-regulation of Id1 and Id3 in bone marrow, presumably by acting on progenitor cells that become mobilized (51). Activation of the mitogen-activated protein kinase (MAPK) pathway by VEGF receptor stimulation could impinge on Id1 and Id3 promoters at the EGR1 site (52). Further study is needed to understand exactly how Id1 and Id3 interact with VEGF to promote the malignant progression in SCLC cells.

The results of this study demonstrated that both Id1 and Id3 are important positive regulators in the tumorigenicity of SCLC and that co-suppression of Id1 and Id3 can significantly inhibit this tumorigenicity *in vitro* and *in vivo*. The results revealed that Id1 and Id3 may promote malignant progression of SCLC cells through facilitating angiogenesis and suppressing apoptosis. These results, in combination with our previous discovery (18), suggested that Id1 and Id3 may not only be diagnostic and prognostic biomarkers, but also valuable targets for designing biologically appropriate strategies for more effective therapy for SCLC.

## MATERIALS AND METHODS

### Cell lines and culture

Variant human SCLC cell line N417 and normal bronchial epithelial cell line Beas-2B were grown in RPMI-1640 (Life Technologies), supplemented with 10% (v/v) FCS (Biosera), 2 mmol/l L-glutamine,100 U/ml penicillin and 100 μg/ml streptomycin (Lonza). Human Umbilical Vein Endothelial Cells (HUVECs) were cultured with EndoGRO reduced-serum medium (Millipore). All cell lines were maintained at 37°C tissue culture incubator in the presence of 5% CO_2_.

### RNA interference

Three 21 nt siRNA sequences targeting Id1 were selected by running the siRNAext program on the website of The Whitehead Institute (http://jura.wi.mit.edu/siRNAext). Nucleotide sequences of three candidate siRNAs against Id1 were as follows:

Sequence 1 for Id1: sense strand 5': CGCCAAGAAUCAUGAAAGU UU; antisence strand 5': ACUUUCAUGAUUCUUGGCG UU;

Sequence 2 for Id1: sense strand 5': CUAGUCACCAGAGACUUUA UU; antisence strand 5': UAAAGUCUCUGGUGACUAG UU

Sequence 3 for Id1: sense strand 5': CGCAUCUUGUGUCGCUGAA UU; antisence strand 5': UUCAGCGACACAAGAUGCG UU”.

To minimize the siRNA off targeting effects, all chosen siRNA were tested with nucleotide BLAST (http://blast.ncbi.nlm.nih.gov/Blast.cgi). The proper siRNA sequence for Id3 was selected in the way described in our previous work (18). A Silencer® Select Negative Control (Life Technologies) was purchased commercially. N417 cells (2×10^5^ per well) were plated in 6-well plates and treated with X-tremeGENE siRNA Transfection Reagent (Roche) and 8 μg Id1-siRNA or negative control. Cells were harvested after 24 and 48 hours respectively and subjected to Western blot analysis. After this transient transfection, the most effective Id1-siRNA sequence which caused the greatest reduction in expression level of target gene was chosen for short hairpin RNAs (shRNAs) construction. Two shRNA inserts consisted of Id1 and Id3 siRNAs respectively were designed using tools online (http://www.sirnawizard.com), and cloned into the psiRNA-DUO vector (InvivoGen) which is capable of expressing two shRNA simultaneously. Two scrambled RNA sequences were cloned into the same plasmid as control. N417 cells were transfected with a vector containing Id1 and Id3 shRNAs or a vector harbored scramble RNA molecules using X-treme GENE HP DNA transfection reagent (Roche). A separate construct containing shRNA against Id1 only was also transfected in parallel. To isolate single colony from the suspended cells, stable transfectants were selected 4 weeks after cells were cultured in a semisolid ClonaCell-TSC (StemCell Technologies) selective medium containing 75 μg/ml Zeocin (Life Technologies).

### Western blot analysis

For immunoblotting, cells were harvested and lysed with a celLytic-M kit (Sigma-Aldrich) following the manufacture's instruction. Samples were separated by 15% SDS-PAGE and transferred to PVDF membrane (Amersham) using a Mini-PROTEAN 3 apparatus (Bio-Rad). Blots were incubated with anti-Id1 antibody (0.5 μg/ml, Abnova), anti-Id3 antibody (1 μg/ml, Abnova) for 1 hour at room temperature or with anti-VEGF antibody (2 μg/ml, Thermo Scientific) overnight at 4°C.

### Invasion assay

Invasiveness of the transfectant cells were tested with Boyden chamber assay using BioCoat™ Growth Factor Reduced (GFR) MatrigelTM Matrix (BD), which was pre-coated with matrigel inserts. Four ×10^4^ cells were plated in the upper chamber in culture medium without FCS. The medium containing 10% FCS was placed in the lower chamber as an attractant. The invasion chamber and control were incubated for 48 hours in a humidified tissue culture incubator at 37°C, 5% CO_2_ atmosphere. The invaded cells which crossed the membrane into the bottom chamber were quantitated by counting three different fields with a cytometer.

### Proliferation assay

Transfectant cells were seeded in a 96-well plate at a density of 5000 cells/100μl culture medium/well. The growth of the cells was measured color metrically every other day for a period of 8 days. On day 2, 4, 6 and 8, the cells were stained respectively with 20 μl CellTiter 96® AQueous One Solution Reagent (Promega).The culture plates were incubated for 4 hours at 37°C in a humidified, 5% CO_2_ atmosphere to allow full color development and the number of cells was calculated according to the absorbent at 490 nm in a spectrophotometer (BioTek).

### Soft agar assay

The anchorage independent growth of the SCLC cells as an indication of cell tumorigenicity was tested in soft agar. The transfectants and N417 cells (1×10^4^) were harvested and seeded in 24-well plates with low melting agarose gel. The plates were incubated in 37°C incubator with 5% CO_2_ and 95% humidity for 4-6 weeks for colony formation. Colonies were stained with 100 μl of MTT (5 mg/ml, Sigma-Aldrich) for 4 hours at 37°C and quantitated with an automated scanner (Oxford Optronix Gelcount).

### Tumorigenicity assay *in vivo*

Tumorigenicity of the transfected SCLC cell lines was tested by s.c. inoculation in 5-week-old male Balb/c nude mice (Harlan). For each nude mouse, 2×10^6^ cells in 200 μl of matrigel/PBS (at a ratio of 1:1) were injected subcutaneously in both flanks. Tumor size was estimated by measuring three dimensions (length, width and depth) of tumors with calipers twice a week. Volumes of tumors were calculated according to the formula: Volume=π/6(H×W)^3/2^ 0.67 (19). The tumor weight was measured by weighing them directly with a balance at autopsy at the end of 3 weeks experimental period. All animal experiments were conducted under UKCCCR guidelines with Home Office Project Licence PPL 40/2270 to Prof Y. Ke.

### Immunohistochemistry

Tumors produced in nude mice were surgically removed at autopsy, fixed in 10% buffered formalin for 24 hours, and embedded in paraffin wax as described in our previous work (20, 21). Tumor sections (5-μm-thick) cut with a microtome (Leica) were placed in coated slides (Leica) and deparaffinized as described previously (18). For immunohistological staining, slides were incubated at room temperature for 1 hour with anti-Id1 antibody (2 μg/ml, Santa Cruz), at 4°C overnight with anti-Id3 antibody (2 μg/ml, Santa Cruz), or at 4°C overnight with anti-VEGF antibody (4 μg/ml, Thermo Scientific). Staining of slides was detected with 3, 3' diaminobenzidine tetrahydrochlorate (DAB) substrate solution (DAKO) which was diluted by 1:50 on each section. All slides were counterstained with hematoxylin (Mayer's) and dehydrated through ethanol and xylene. The intensity of the staining was observed under microscope and scanned with a ScanScope (Aperio Technologies) image scanner. Nucleic staining was scored by combining the stain intensity with percentage of cells stained. The intensity of staining was assessed by examining approximately 10 fields at 40× magnifications and was divided into 4 categories (no stain = 0, weakly stained = 1, moderately stained = 2, strongly stained = 3). The scores of percentage staining were also classified into 4 categories according to the percentages of the stained cells (0% = 0, 1–30% = 1, 31–69% = 2, 70−100% = 3). The final score (with points from 0-9) of immunohistological staining of a tissue section was obtained by multiplying the intensity score with the percentage score. Thus the nuclear staining was finally rated as follows: 0, negative staining; 1-3, weak staining; 4-6, moderate staining; 7-9, strong staining. Cytoplasmic staining was categorized as negative, weak, moderate and strong staining according to the intensities. The classification for VEGF staining was slightly different as follows: negative, very weak, weak, and moderate staining.

### Flow cytometry

In this study, the apoptosis of SCLC cells was induced by cisplatin which is a first-line treatment drug used in SCLC chemotherapy. After different doses (0, 25, 50, 75, 100 and 125 μM) of cisplatin (Sigma) were applied to each cultured cell line, the cells were incubated in 37°C for approximately 24 hours. Those percentage of cells undergoing apoptosis was determined by the fluorescence intensity of Annexin V-PE which was measured with a FACS Calibur cytometer (BD biosciences) using a phycoerythrin emission signal detector (FL2 channel).

### VEGF ELISA assay

Transfected cells were seeded onto a 6-well plate at a density of 1×10^6^ and cultured for 24 hours. Conditioned medium (100 µl) from each cell line was removed and the VEGF presented in the medium was examined with human VEGF ELISA kit (RayBio) following the manufactory's instruction. VEGF protein concentration in the medium was determined color metrically using a micro-plate reader (BioTek) with a wavelength of 450 nm.

### Angiogenesis assay *in vitro*

Biological activity of the secreted VEGF was tested by measuring its ability to stimulate the HUVECs to form blood vessels. Fifty μl ECMatrix™ Gel (*In Vitro* Angiogenesis Assay Kit, Merck Millipore) was placed in each well of a sterile pre-cooled 96-well plate. HUVECs (5×10^3^) in 100 μl EndoGRO reduced-serum medium were treated with 100 μl conditioned medium obtained from each transfected cell culture (preparation protocol has been described in VEGF ELISA assay). Same number of HUVECs was incubated with human VEGF antibody to assess whether the angiogenesis activity was caused by VEGF only. HUVECs incubated with 10 ng/ml recombinant human VEGF protein (RayBio) and those incubated in growth medium only were set as the positive control and negative control respectively. The 96-well plate was incubated at 37 °C, 5% CO_2_ for 6 hours. Once vascular structure was visualized, the reaction were stained with 50 μl of MTT (5 mg/ml) for 10 minutes and quantified under an inverted microscope with 40× magnification.
